# Use of Moisturizers and Lubricants for Vulvovaginal Atrophy

**DOI:** 10.3389/frph.2021.781353

**Published:** 2021-12-23

**Authors:** Ayane Cristine Alves Sarmento, Márcia Farina Kamilos, Ana Paula Ferreira Costa, Pedro Vieira-Baptista, José Eleutério, Ana Katherine Gonçalves

**Affiliations:** ^1^Health Sciences Postgraduate Program, Federal University of Rio Grande Do Norte (UFRN), Natal, Brazil; ^2^Department of Gynecology, Hospital Heliópolis, São Paulo, Brazil; ^3^Lower Genital Tract Unit, Centro Hospitalar de São João, Porto, Portugal; ^4^Hospital Lusíadas Porto, Porto, Portugal; ^5^Department of Obstetrics and Gynecology, Federal University of Ceará, Fortaleza, Brazil; ^6^Department of Obstetrics and Gynaecology, Federal University of Rio Grande Do Norte (UFRN), Natal, Brazil

**Keywords:** menopause, vulvovaginal atrophy, moisturizers, lubricants, non-hormonal treatment

## Abstract

The estrogen decrease in postmenopausal women results in functional and anatomical changes in the genitourinary tract. The most prevalent and bothersome symptoms are vaginal dryness, dyspareunia, and reduced lubrication, which can significantly affect the quality of life of these women, principally those who are sexually active. Hormonal therapy with local estrogens is generally considered the “gold standard.” However, there are cases in which there are clinical concerns about its use or women opt for non-hormonal options. Thus, safe and effective non-hormonal options are needed to improve symptoms in these women. Moisturizers and lubricants are first-line therapy for breast cancer survivors.

## Introduction

Vulvovaginal atrophy (VVA) is one of the most striking and acknowledged manifestations of the so-called “genitourinary syndrome of menopause” (GSM), and can get worse if not treated (1, 2). VVA is associated with dryness, dyspareunia, sexual dysfunction, nocturia, dysuria, and recurrent urinary infection (3, 4). The intensity of symptoms is related to the time elapsed since menopause and the frequency of sexual intercourse (3, 4). The main objective of treatment is symptomatic relief and depends on factors such as age, overall health, presence of other climacteric symptoms, and health risks (5). Many women try to alleviate postmenopausal symptoms with over-the-counter products, like moisturizers or lubricants, while others seek the help of a gynecologist or other health care professionals (6).

Treatment options include both local and systemic hormonal or non-hormonal options. Topical estrogens, considered the gold standard for the treatment of VVA, are the most utilized and effective treatments (5–8). They can be administered in various formulations (tablets, creams, suppositories, and pessaries) and dosages (5–9). However, studies have shown that topical formulations can also lead to increase in the serum estrogen levels within considered normal limits for menopause. Therefore, despite some evidence of safety, caution is recommended in those with a history of estrogen-sensitive endometrial or breast cancers (7–9).

However, non-pharmacological treatments can also be beneficial and are considered the therapy first-line for women with contraindications or fear of hormonal treatments. Beyond lifestyle changes, non-pharmacological treatments include moisturizers, lubricants, phytoestrogens, and laser (5–10). Simple therapies that can reduce the negative impacts of menopause and preserve healthier condition. The approach must be holistic, considering the different aspects of the human dimension: physical, emotional, mental and socioeconomic. Investing in self-care, with simple measures such as not smoking, ensuring adequate sleep, adequate sun exposure, is good at any stage of life, especially in the climacteric, where small changes, can translate into significant improvement (1, 8, 10).

Smoking enhances estrogens metabolism and, consequently, increases vaginal atrophy (7). A high body mass index height (>27 kg/m^2^) and sedentary also are associated with the presence of vaginal symptoms, probably due to a lower vascular supply to the genitourinary area (11). Sexual intercourse and/or masturbation are also beneficial, improving elasticity, lubrication, and vascularization, and promoting improvement of symptoms such as dyspareunia (12).

Despite being less effective than hormonal treatments, some women and physician's opt for non-hormonal treatments, such as moisturizers and lubricants, as the first option of treatment to alleviate vulvovaginal symptoms (13).

## Vaginal Moisturizers

Vaginal moisturizers should be applied regularly-generally, 1–3 times a week. They act through adherence to the vaginal mucosa, promoting hydration that stimulates lubrication. In addition, tissue integrity, elasticity, and pliability are improved. Moisturizers are constituted by water and other substances such as hyaluronic acid or polycarbophil (14, 15).

Vaginal moisturizers are used mostly for symptomatic treatment, especially of vaginal dryness. Some substances, such as hyaluronic acid, also facilitate cell migration during inflammation states and the cellular repair process, thus having a role in maintaining tissue integrity. A study of efficacy comparing these to local estrogenic preparations has found similar improvements for the outcomes of vaginal dryness and pH (16, 17). Moisturizers are an option for women with mild to moderate symptoms, although eventually require hormonal therapies (10, 18).

## Lubricants

Lubricants can be water, silicone, or oil-based products that are not skin or mucosa-absorbed. They have immediate action and promote temporary relief of vaginal dryness and pain during sexual activity; hence, they are helpful for women who complain about vaginal dryness (19, 20). Water-soluble lubricants, in general, are associated with fewer genital side effects than silicone ones, such as mucosal irritation (3, 13, 21). Studies conducted *in vitro* and *in vivo* animals showed that water-based lubricants promote changes in the vaginal environment and mucosa, may be caused to toxic effects, and increase the transmission of sexually transmitted pathogens, such as HIV (3, 22).

Recently, was issued an “Advisory Note” by The World Health Organization (WHO) about the technical requirements for lubricants, especially when used in addition to condoms (23). The requisites included are: osmolality values of 380 mOsm/kg or lower are desirable. However, values as high as 1,200 mOsm/kg have been considered tolerable provisionally. In addition, intrinsic ingredient toxicity and pH. Alterations from the normal vaginal pH in the healthy adult, between 3.5 and 4.5, are considered as potentially prejudicial (3, 21–23).

The use of lubricants during intercourse may reduce the irritation caused by the friction on the tissue. Recently, lubricants products have been developed to avoid altering the physical properties of the condom, identical pH, and osmolarity of semen and cervical mucus do not alter the viability and motility of the sperm (3, 21–25).

## Conclusion

Recently, various treatment modalities have developed to control the still is condition's bothersome and aggravating menopausal symptoms. However, first-line therapy still consists of non-hormonal formulations such as lifestyle changes, lubricants, and moisturizers. The latter helps relieve the symptoms of VVA. For this reason, are commonly used daily and for sexual intercourse and are especially useful in women who cannot use topical or systemic estrogens. Lubricants and moisturizers present good results and minimal side effects and should be chosen for those who are body-similar in terms of pH and osmolality to reduce the chances of endothelial irritation and side effects.

## Author Contributions

ACAS and AKG conceived and designed the study. ACAS, AKG, APFC, and PV-B drafted and revised the article where appropriate. ACAS and APFC prepared the tables. AKG, PV-B, JEJ, and MK carried out the final revision of the manuscript. All authors contributed to the article and approved the submitted version.

## Conflict of Interest

The authors declare that the research was conducted in the absence of any commercial or financial relationships that could be construed as a potential conflict of interest.

## Publisher's Note

All claims expressed in this article are solely those of the authors and do not necessarily represent those of their affiliated organizations, or those of the publisher, the editors and the reviewers. Any product that may be evaluated in this article, or claim that may be made by its manufacturer, is not guaranteed or endorsed by the publisher.
